# The impacts of the COVID-19 pandemic on birth satisfaction and birth experiences in Russian women

**DOI:** 10.3389/fgwh.2022.1040879

**Published:** 2022-12-21

**Authors:** Anna Suarez, Vera Yakupova

**Affiliations:** Department of Psychology, Lomonosov Moscow State University, Moscow, Russia

**Keywords:** birth satisfaction, COVID-19 pandemic, birth experience, mode of birth, support during labour, antenatal education, medical interventions, childbirth plan

## Abstract

**Background:**

Women's satisfaction with their childbirth experiences has significant impacts on their health and the health of their children. Recently, childbirth and maternity care systems have been disrupted by the COVID-19 pandemic. This study aimed to investigate the association of birth satisfaction with mode of birth, medical interventions, support during labour, type of childbirth healthcare plan and antenatal education in the context of the COVID-19 pandemic in Russia.

**Methods:**

1,645 Russian women who gave birth during the first year of the COVID-19 pandemic and 611 matched controls who gave birth in the previous year participated in an anonymous Internet survey about their childbirth experience. The survey included questions regarding women's demographic and obstetric characteristics as well as their childbirth experiences. Birth satisfaction was measured using the Birth Satisfaction Scale Revised Indicator (BSS-RI).

**Results:**

Birth satisfaction scores did not show notable changes before and during the pandemic (Pearson Chi-square = 19.7, *p* = 0.22). Women had lower BSS-RI scores if they tested positive for COVID-19 during labour (*F* = 9.18, *p* = 0.002), but not during pregnancy or postpartum (*p* > 0.32). In both cohorts women who had vaginal births rated birth satisfaction higher than those who had caesarean births. The more medical interventions there were, the lower were the BSS-RI scores (*B* = −0.234, 95% CI: −0.760; −0.506, *p* < 0.001), but only during the pandemic. Birth satisfaction was higher if women had a support person present during labour (*F* > 7.44, *p* < 0.001), which was not possible for over 70% of participants during the pandemic. In both cohorts birth satisfaction was associated with the childbirth healthcare plan (*F* > 5.27, *p* < 0.001), but not with antenatal education (*F* < 0.15, *p* > 0.43).

**Conclusions:**

Our study highlights the significant impacts of the COVID-19 pandemic on the birth experiences of Russian women. Sustaining the rights of women to informed decisions during labour, respect for their preferred childbirth healthcare plan, presence of the birth team of choice and professional support for home birth are essential for higher birth satisfaction and better health outcomes for mothers and their infants.

## 1. Introduction

Women's satisfaction with their childbirth experiences has significant impacts on their health. A negative birth experience is associated with higher rates of postpartum depression (1), posttraumatic stress disorder (PTSD) (2), fear of childbirth, and increased desire for an elective caesarean birth (CB) in future pregnancies (3, 4). Furthermore, it may have long-term consequences for interactions with their children, such as compromised mother-infant bonding (5–7) and lower rates of exclusive breastfeeding (8). This reduces maternal quality of life, weakens mother-child relationships, and can lead to abnormal physical, psychological and emotional development of the child (9).

Sadly, worldwide reproductive health services have conventionally focused their efforts and resources on reducing perinatal mortality and have paid less attention to the psychological aspects of women's childbirth experiences (10, 11). However, there is a trend of acknowledging the importance of maternal perinatal mental health and women's satisfaction with labour, which is reflected in both the 2016 World Health Organisation (WHO) guidelines for antenatal care (12) and the 2018 WHO guidelines for intrapartum care (13), which highlight the importance of having a positive childbirth experience to the birthing persons.

Birth satisfaction has been linked to various obstetric, sociodemographic, and psychological factors (14, 15). By and large, the most frequently studied factors are birth-related, such as type of childbirth healthcare plan and mode of birth. Consistent research documents that women who have birth experience outside of the hospital, e.g., in birth centres or at home, rate those experiences more favourably compared to in-hospital births (16). Contrarily, the reports on the influence of mode of birth on women's experiences of childbirth are conflicting. While a number of studies indicate that there is no direct association between mode of birth and satisfaction with childbirth (17, 18), there is convincing evidence that emergency CBs significantly increase the risks of negative birth experience compared to other modes of birth (19, 20). In a Swedish population-based cohort study with over 16,000 participants, emergency CB was the strongest predictor of reporting dissatisfaction with childbirth, with no significant association for elective CB (19). Instrumental vaginal delivery was also a risk factor for dissatisfaction with childbirth compared to a normal vaginal birth (19).

The association between women's levels of satisfaction with their birth experiences and the use of epidural analgesia is also debated. In a Swedish study Ulfsdottir and colleagues found the use of epidural analgesia to be a risk factor for a negative childbirth experience (21). This finding is supported by the results from the study of Italian primiparous women (17). Contrarily, a systematic review shows no significant association between maternal satisfaction and epidural analgesia (22).

While obstetric factors are most studied in relation to birth experience, in her systematic review, Ellen Hodnett indicates that overall, biomedical interventions did not affect women's experiences of childbirth as much as the attitudes and behaviours of the healthcare providers (22). Indeed, several studies have shown that the amount of support from the midwife or other caregivers and the provider-patient relationship have vastly contributed to women's perception of their childbirth experiences (15, 22, 23). Hodnett and colleagues further show that continuous support during labour, particularly when provided by a woman who was neither part of the hospital staff nor the woman's social network, e.g., a doula, has clinically meaningful benefits for women and their children (24). Contrarily, a systematic review and meta-analysis that included 20 trials with over 22,000 participants from 12 countries demonstrated that labour support from someone in a close relationship with the birthing person rather than a hired companion was promoting more positive birth experiences (23). This review and meta-analysis of prenatal and intrapartum interventions further showed that birth preparedness was a successful strategy for improving the experience of birth and increasing birth satisfaction (23). Attending childbirth preparation classes may help achieve birth preparedness and, indeed, a study from Iran shows that women who regularly attended such classes scored higher on a birth satisfaction scale (25). Conversely, a study from Alaska did not find that attendance at a childbirth class had an impact on satisfaction with the birth process, while it did minimise the use of biomedical interventions (26).

Recently, another factor came into play, which has had significant impacts on women's birth satisfaction. In 2020 childbirth and maternity care systems around the world were disrupted by the COVID-19 pandemic (27). Protocols changed rapidly, healthcare facilities were rearranged, and care routines were disarrayed. As the risks of COVID-19 infection for mother and baby were initially not well understood, multiple restrictive measures to mitigate virus transmission were implemented in hospitals and other maternity care facilities worldwide (27). Necessary alterations to antenatal care such as cancellation of appointments and in-person birth preparation classes as well as termination of hospital tours, caused women to feel less prepared for birth and more stressed and anxious (28). In a prospective cohort study of 2,341 U.S. women, maternal stress about feeling unprepared for birth due to the pandemic and restrictions on companions during birth independently predicted lower birth satisfaction (29). Furthermore, unpreparedness stress due to the pandemic was indirectly influencing birth satisfaction through a mediation process *via* association with more biomedicalised birth and greater incongruence with women's birth plan (29). Women who gave birth during the peak of the pandemic and those who tested positive for COVID-19 during the hospital admission process, had lower birth satisfaction and gave a worse rating of the quality of care received both in U.S (30). and Spanish (31) studies.

In Russia, in January-February 2020 the government introduced multiple measures to contain the pandemic which varied across the regions (32), with the majority of maternity care hospitals restricting the possibility for any support person to attend birth (33). Moreover, there were recommendations to separate the mother and her baby right after birth in case of the mother's positive test for COVID-19 (33). A previous study of maternal mental health and changes in the childbirth context due to the COVID-19 pandemic in Russia showed that these measures led to an immense lack of support during and after labour, with less than 30% of women being accompanied during childbirth (34). Moreover, women reported more instances of obstetric violence and emotional abuse during the pandemic compared to pre-pandemic times (34). Altogether these changes may have contributed to negative birth experiences and affected women's satisfaction with their childbirth experience. However, to our knowledge there are no studies looking into the question of birth satisfaction in Russia and changes that may have affected it during the COVID-19 pandemic.

It is important to note that there are certain characteristics of the maternity healthcare system in Russia, which distinguish it from other countries and may have affected birth satisfaction prior to COVID-19 pandemic. According to sociological studies, there are two kinds of approaches to obstetric care in the Russian maternity healthcare system, with a conservative soviet approach on the one hand and a modern evidence- and ethics-based approach on the other (35). Conservative soviet approach is characterised by a paternalistic style of communication, outdated and often routine medical practices with lack of ethical concern and respect for individual needs and interests of the pregnant women and their partners (36). Moreover, while the presence of a birth partner is a legal right since 2012 in Russia (35), small maternity care hospitals, particularly in the remote Russian regions, can still restrict the birth partner's presence during labour due to the absence of individual wards. Furthermore, in the majority of hospitals the opportunity for continuous individual support by a doula or a privately hired midwife is not available as part of state healthcare and is possible only if the woman pays both for a doula or private midwife service and for the contract with the hospital in order to have this option included in her childbirth plan. However, not all maternity hospitals allow such services as women's right to have a doula or other support person who is unrelated to her or the baby during labour is not guaranteed by the law (37). Thus, birth culture in Russia has been complex and required planning and investment for the women's wishes and childbirth healthcare plan to be respected before the pandemic.

However, despite the persistence of these conservative soviet practices, gradually many healthcare providers choose modern evidence-based approaches (38) and follow the WHO guidelines for intrapartum care (13). This trend is also reflected in the new recommendations for obstetric and gynaecology care of the Ministry of Health of the Russian Federation (39). There is evidence that in the past ten years more women have been choosing to give birth with at least one support person present at labour and birth, birth partners have been more involved, and doula services have become more popular, with an official Association for professional doulas established in 2015 (38, 40). However, many of these trends were interrupted by the COVID-19 related measures and the maternity healthcare system returned to more familiar conservative practices (33).

Therefore, the main objective of this study was to investigate the association between women's birth satisfaction and obstetric and other factors in the context of the COVID-19 pandemic. Namely, we examined whether birth satisfaction was associated with mode of birth and biomedical interventions as well as with support during labour, types of childbirth healthcare plan and childbirth preparation. Furthermore, we tested whether or not there were changes in those associations due to COVID-19 itself and related restrictive measures, as we now have a unique opportunity to compare the data collected from women who gave birth during the first year of the COVID-19 pandemic (February 2020–March 2021) and matched controls who gave birth one year prior (January 2019–February 2020). Finally, we examined whether birth satisfaction was associated with having COVID-19 diagnosis before, during or after delivery.

### 2. Materials and methods

### 2.1. Procedure and participants

During the period from January to February 2020 and February to March 2021 women were invited to take part in the study *via* perinatal education classes and specialised online and offline communities for new parents. 611 mothers of infants aged 0–13 months (M = 6.37) participated in the study before the pandemic, and 1,645 mothers of infants aged 0–13 months (*M* = 6.93) completed the online survey during the pandemic. All respondents provided informed consent to participate in the study. The inclusion criteria were respondent's age of 18 years and over, ability to read and write in Russian, and having given birth to a live-born child no longer than 14 months prior to the study.

### 2.2. Measures

#### 2.2.1. The demographic, pregnancy and childbirth experience survey

The survey for both cohorts included questions regarding the participants' age at the time of childbirth, education (basic school education/vocational education/higher education), place of childbirth (Moscow and capital region/Other city in Russia with population >1 million/Other city in Russia with population <1 million/Post-Soviet States/Other), and marital status (married/cohabiting with partner/single). Information about the type of the childbirth healthcare plan was collected (childbirth in a specialised maternity care hospital under state insurance/childbirth in a specialised maternity care hospital with a paid contract and option for a birth team of choice/home birth). Respondents also provided information regarding obstetric characteristics such as parity, gestational age at birth, time since birth, and mode of birth (vaginal/assisted vaginal/emergency CB/planned CB).

The participants were also asked to report whether there were any biomedical interventions during labour and what type of interventions were administered (epidural analgesia/episiotomy/amniotomy/synthetic oxytocin/other).

Further information about the sources of support during labour was collected (none/partner/doula or private midwife/partner + doula or private midwife) and antenatal education type (none/self-education/educational courses/mixed educational strategies, where participants chose several sources for childbirth preparation).

The survey also included questions about the COVID-19 diagnosis: whether the respondents themselves or any of their family members were diagnosed with COVID-19 during pregnancy, at the moment of delivery or postpartum.

#### 2.2.2. Birth satisfaction scale revised indicator (BSS-RI)

We used the Birth Satisfaction Scale Revised Indicator (BSS-RI) (41) to assess the levels of birth satisfaction (42). It is a short 6-item self-report questionnaire to assess birth satisfaction where the subscales represent the level of stress and anxiety, feeling of control, and caregivers' support. A 3-point Likert scale is used for each question (range 0–2, where 0 means “no”, 1 means “partly” and 2 means “yes”). Minimum score is 0, maximum score is 12. Higher scores represent greater birth satisfaction. The Russian version in the current study showed high validity (Cronbach's *α* = 0.805).

#### 2.2.3. Covariates

All the analyses were controlled for maternal age at the time of childbirth, level of education, family status, time after childbirth, gestational age, parity and place of childbirth.

### 2.3. Statistical analysis

Spearman's correlation coefficient was used to estimate the relationship between BSS-RI scores and the covariates.

We explored the association between the BSS-RI scores and birth experience factors (mode of birth, type of childbirth healthcare plan, type of support during labour, childbirth preparation type) using generalised linear models.

Multiple linear regression analysis examined the association between BSSR-RI scores and age at testing, gestational age and parity.

Pearson Chi-square tests were performed to compare the demographic and birth experience characteristics between the first cohort (before the pandemic) and the follow-up (during the pandemic).

All analyses were performed using IBM SPSS 25 software (43).

## 3. Results

Demographic, obstetric and childbirth characteristics for participants from the two stages of the study before and during the COVID-19 pandemic are presented in Table 1.

**Table 1:** Characteristics of the sample.

Characteristics		Women who gave birth before the pandemic (*n *= 611)			Women who gave birth during the pandemic (*n* = 1645)			*P*-value
		Mean/*N*	SD/%	Range	Mean/*N*	SD/%	Range	
Age at testing (years)		31.17	4.54	18–45	30.98	4.42	19–50	0.39
Education	Upper Secondary/College	57	9.3%		135	8.2%		0.40
	Tertiary/University	554	90.7%		1510	91.8%		
Family Status	Married	559	91.5%		1547	94.0%		0.024
	Cohabiting With a Partner	33	5.4%		74	4.5%		
	Single	19	3.1%		24	1.5%		
Time After the Childbirth (Months)		6.37	3.42	0.2–12	6.93	3.30	0–14	<0.001
Gestational Age		39.47	1.67	28.0–43.0	39.40	2.04	0–43.0	0.45
Mode of birth	Vaginal	442	72.5%		1142	69.4%		<0.001
	Assisted vaginal	30	4.9%		41	2.5%		
	Emergency CB	86	14.1%		193	11.7%		
	Planned CB	52	8.5%		269	16.4%		
Place of Birth	Moscow and Capital Region	224	36.7%		403	24.6%		<0.001
	Other city in Russia with population >1 million	262	43%		583	35.6%		
	Other city in Russia with population < 1 million				461	28.2%		
	CIS Countries	42	6.9%		98	6.0%		
	Europe/USA/Other	82	13.4%		91	5.6%		
Parity	1	359	58.8%		971	59%		0.022
	2	173	28.3%		522	31.8%		
	3+	79	12.9%		152	9.2%		
Type of childbirth plan	Childbirth in a specialised maternity care hospital under state insurance	344	56.3%		1020	62.0%		0.020
	Childbirth in a specialised maternity care hospital with a contract for a hospital or medical team of choice	250	40.9%		598	36.4%		
	Home birth	17	2.8%		27	1.6%		
Had at least one medical intervention during labour		517	84.6%		1386	84.3%		0.90
Number of medical interventions		1.62	1.19		1.57	1.19		0.40
Types of medical interventions	Epidural analgesia	244	39.9%		655	39.8%		0.96
	Episiotomy	116	19.0%		332	20.2%		0.55
	Amniotomy	279	45.7%		687	41.8%		0.10
	Synthetic oxytocin	230	37.6%		541	32.9%		0.036
Types of antenatal education	None	153	25%		410	24.9%		<0.001
	Self-education	226	37%		744	45.2%		
	Educational courses	101	16.5%		250	15.2%		
	Mixed type (more than one type of education)	131	21.4%		241	14.7%		
Support person at labour (yes)		354	57.9%		443	27.0%		<0.001
Mode of birth support	No support	257	42.1%		1200	73.0%		<0.001
	Partner	217	35.5%		199	12.1%		
	Doula/Private midwife	74	12.1%		178	10.8%		
	Partner + doula/private midwife	63	10.3%		66	4.0%		
BSS-RI		7.81	3.02	0–12	7.63	3.21	0–12	0.22
Confirmed Covid-19	During pregnancy	NA	NA		111	4.9%		NA
	During labor	NA	NA		35	1.6%		NA
	Postpartum	NA	NA		121	5.4%		NA

*P*-values come from Pearson Chi-square (for nominal variables) and independent *t*-test (for continuous variables) statistics comparing the cohorts before (*N* = 611) and during pandemic (*N* = 1645). CS for CB. BSS-RI stands for the Birth Satisfaction Scale Revised Indicator.

Birth satisfaction scores did not show notable changes before and during the pandemic (Pearson Chi-square = 19.7, *p* = 0.22).

Among the covariates before the pandemic birth satisfaction positively correlated with parity (*B* = 0.17, 95% CI: 0.30; 0.92, *p* < 0.001) and negatively with age (*B* = −0.10, 95% CI: −0.12; −0.009, *p* = 0.024). During the pandemic there was no correlation with age, while the association with parity remained statistically significant (*B* = 0.17, 95% CI: 0.53; 0.98, *p* < 0.001). Furthermore, in the follow-up study birth satisfaction scores were correlated with gestational age at birth (*B* = 0.54, 95% CI: 0.009; 0.16, *p* = 0.028), with no such association before the pandemic (*B* = 0.03, 95% CI: −0.10; 0.19, *p* = 0.54). Contrarily, there was a significant association between the place of birth and BSS-RI scores before the pandemic (*F* = 2.92, *p* = 0.033), but not during the pandemic (*F* = 2.16, *p* = 0.070). There were no significant associations with other covariates either before or during the pandemic (*p*-values for all >0.57; data not shown).

### 3.1. Mode of birth, biomedical interventions, and birth satisfaction

During the pandemic the frequencies of different modes of birth have changed (Pearson Chi-square = 147.06, *p* < 0.001). There were fewer emergency CBs (Pearson Chi-square = 112.77, *p* < 0.001) and more planned CBs (Pearson Chi-square = 22.33, *p* < 0.001) during the pandemic in comparison to pre-pandemic times. Furthermore, the frequency of assisted vaginal births also decreased significantly during the pandemic (Pearson Chi-square = 23.18, *p* < 0.001).

After adjustment for covariates there was a significant association between BSS-RI scores and mode of birth both before (*F* = 21.3, *p* < 0.001) and during the pandemic (*F* = 60.8, *p* < 0.001). Table 2 shows that in both cohorts women who had vaginal births rated birth satisfaction higher than those who had CBs (Table 2). However, before the pandemic birth satisfaction was lowest only among those who had emergency CBs, while during the pandemic women who had any type of CB had significantly lower scores than those who gave birth vaginally.

**Table 2:** The mean BSS-RI scores for different characteristics of birth experience in the cohorts of women who gave birth before or during the COVID-19 pandemic.

Characteristics		Women Gave Birth Before Pandemic (*n* = 611)		Women Gave Birth During Pandemic (*n* = 1645)	
		Mean BSS-RI	SD	Mean BSS-RI	SD
Place of Birth	Moscow and Capital Region	7.99*	3.03	7.94	3.16
	Other city in Russia with population >1 million	7.79*	3.06	7.44	3.28
	Other city in Russia with population < 1 million			7.57	3.21
	CIS Countries	6.55*	2.81	7.35	3.23
	Europe/USA/Other	8.07*	2.88	8.12	3.29
Delivery Mode	Vaginal	8.40**	2.78	8.27**	3.06
	Emergency CB	5.58**	2.95	5.73**	3.72
	Planned CB	6.96**	3.09	5.43**	2.92
	Assisted vaginal	6.57**	3.25	7.25**	2.82
Medical interventions	At least one intervention	7.81	3.04	7.45**	3.26
	None	7.82	2.94	8.57**	2.77
	Amniotomy	8.02	3.02	7.60	3.34
	No amniotomy	7.63	3.02	7.65	3.12
	Epidural analgesia	7.68	3.01	6.97**	3.20
	No epidural analgesia	7.90	3.03	8.07**	6.97
	Episiotomy	7.80	3.22	7.11*	3.32
	No episiotomy	7.81	2.98	7.76**	3.17
	Synthetic oxytocin	7.83	3.13	7.12**	3.29
	No synthetic oxytocin	7.79	2.96	7.88**	3.15
Type of childbirth plan	Childbirth in a specialised maternity care hospital under state insurance	7.50**	3.00	7.26**	3.32
	Childbirth in a specialised maternity care hospital with a contract for a hospital or medical team of choice	8.14**	3.00	8.18**	2.95
	Home birth	9.18**	3.04	9.30**	2.49
Types of antenatal education	None	6.67	1.52	7.83	3.17
	Self-education	7.89	3.01	7.48	3.24
	Educational courses	7.71	3.01	7.78	3.04
	Mixed type (more than one type of education)	7.87	3.07	7.59	3.36
Support person during labour (yes)	No support	7.32**	2.99	7.32**	3.31
Mode of birth support	Partner	7.74**	3.13	8.24**	2.83
	Doula/Private midwife	8.93**	2.64	8.67**	2.61
	Partner + doula/private midwife	8.68**	2.70	8.52**	2.96
Confirmed Covid-19	During pregnancy	NA	NA	7.44**	3.02
	During labour	NA	NA	5.86**	3.30
	Postpartum	NA	NA	6.88**	3.20

BSS-RI stands for the Birth Satisfaction Scale Revised Indicator, score range is 0–12; CS stands for caesarean birth.

* *p* < 0.05.

** *p* < 0.001.

The frequency of biomedical interventions during labour virtually remained the same before and during the pandemic (Table 1). Table 1 further shows that the frequencies of epidural use, episiotomy, and amniotomy did not change during the pandemic in comparison to pre-pandemic times (*p*-values for all >0.10), while there was a slight decrease in the use of synthetic oxytocin during the pandemic (Pearson Chi-square = 4.48, *p* = 0.036). The more biomedical interventions there were, the lower were the BSS-RI scores (*B* = −0.234, 95% CI: −0.76;-0.51, *p* < 0.001). However, this effect was notable only during the pandemic, with no such association before the pandemic (*B* = 0.10, 95% CI: −0.18; 0.23, *p* = 0.81). Table 2 shows that women who had epidural analgesia, episiotomy or the use of synthetic oxytocin during labour had lower BSS-RI scores, but only during the pandemic.

### 3.2. Support during labour and birth satisfaction

During the pandemic, labour support has changed significantly (Pearson Chi-square = 230.3, *p* < 0.001). Table 1 shows that before the pandemic 42.1% of women gave birth with no support, while during the pandemic this number increased to 73% (Pearson Chi-square = 187.4, *p* < 0.001).

After adjustment for covariates, birth satisfaction was significantly associated with the mode of support during labour both before (*F* = 7.44, *p* < 0.001) and during the pandemic (*F* = 13.09, *p* < 0.001). Table 2 shows that women who gave birth with a private midwife or a doula had the highest mean scores of birth satisfaction in both cohorts.

### 3.3. Type of childbirth healthcare plan and birth satisfaction

Table 1 shows that during the pandemic the participants more often gave birth in a specialised maternity care hospital under state insurance rather than with a contract for a hospital or medical team of choice or at home (Pearson Chi-square = 7.84, *p* = 0.020).

After adjustment for covariates, the type of childbirth halthcare plan was significantly associated with birth satisfaction both before (*F* = 5.27, *p* < 0.001) and during the pandemic (*F* = 18.25, *p* < 0.001). Women who gave birth at home had the highest mean BSS-RI scores in both cohorts (Table 2).

### 3.4. Antenatal education and birth satisfaction

During the pandemic the types of antenatal education changed significantly (Pearson Chi-square = 20.0, *p* < 0.001). Table 1 shows that 45.2% of participants who prepared for childbirth *via* self-education during the pandemic in comparison to 37% of women before the pandemic (Pearson Chi-square = 12.3, *p* < 0.001). The number of participants with a mixed type of antenatal education also increased (Pearson Chi-square = 15.2, *p* < 0.001).

After adjustment for covariates there was no association between BSS-RI scores and the antenatal education type before (F = 0.92, *p* = 0.43) or during the pandemic (*F* = 0.15, *p* = 0.70).

### 3.5. COVID-19 diagnosis and birth satisfaction

Table 1 shows that there were 111 (4.9%) participants who had a test-confirmed case of COVID-19 infection during pregnancy, 35 (1.6%) participants who had it in the hospital while giving birth, and 121 (5.4%) women who reported contracting the infection after giving birth.

After adjustment for covariates, having a confirmed COVID-19 diagnosis was associated with lower BSS-RI scores, if tested positive in the hospital during labour (*F* = 9.18, *p* = 0.002), but not during pregnancy or the postpartum period (*p*-values >0.32).

## 4. Discussion

Our study provides new evidence that the COVID-19 pandemic has had a significant impact on women's birth experiences in Russia due to the alterations in the maternity healthcare system.

In contrast to the findings in the Spanish (31) and American (30) samples, where there was a significant decrease in women's birth satisfaction during the pandemic, there were no such differences in our study. This may be due to the still prevalent and socially approved belief that childbirth may be considered satisfactory as long as the baby is fine, regardless of women's subjective experiences (44). Furthermore, our results are in line with the study from Northern Italy with no significant differences in mothers' satisfaction with their childbirth experience, which the authors partially attributed to possible association with the timing of participants' recruitment (45). Indeed, in our sample women had experiences of giving birth between February 2020 and March 2021, when the conditions in the birth hospitals and COVID-19 related measures were rapidly changing and, additionally, varied among different regions, thus, possibly smoothing the differences in birth experiences in the initial and later, more stabilised stages of the pandemic.

However, similar to the reports from New York City (30), our data confirm that a positive COVID-19 test at the hospital during labour, but not before or after childbirth, is significantly associated with lower birth satisfaction. This may be explained by the fact that there were multiple reports of women who tested positive for COVID-19 facing lack of medical support and separation from their infant right after birth both in Russia (33) and globally (46, 47). This finding supports the argument that COVID-19 testing and other similar measures must be implemented with concern for potential healthcare discrimination and stigma (30).

According to our results, during the pandemic the country of childbirth became less relevant for the birth experience of Russian women, which may be due to the fact that the stress associated with the COVID-19 outbreak and the restrictive measures to limit its spreading, although varied in timing and extent, were shared globally. The place of birth and type of childbirth healthcare plan, on the other hand, remained significant, with home births being associated with the highest birth satisfaction scores both before and during the pandemic. Our results correspond with the previous research, documenting that births outside the hospital tend to be perceived more positively (16). In Russia, medical assistance during home birth is illegal, therefore, this type of childbirth healthcare plan is risky and marginalised (37). Contrary to the data from other countries (48–50), in Russia we documented no increase in home birth rates: in both cohorts home birth rates were below 3% of the sample, which confirms their marginalised status. However, with the proper legislation and professional support, home birth may be a potential avenue to improving women's birth experience.

Our results further indicate that giving birth with a paid contract, i.e., when birth takes place in the state hospital, but there are certain privileges, such as having a birth team of choice and a private chamber, may be a better alternative to regular hospital birth as women reported higher birth satisfaction in such settings (35). Sadly, the majority of participants both before and during the pandemic still gave birth under state health insurance, which was associated with the lowest birth satisfaction scores. This may be explained by the lack of support and privacy, factors that are strongly associated with birth satisfaction (22–24). Thus, availability of sufficient support from the clinicians and the birth team of choice for all groups of patients, regardless of their economic status, is extremely important.

While there is evidence, documenting poor perinatal health care and lower hospital availability during the pandemic (48, 51, 52), our data suggest that there was no decrease in hospital and biomedical care access during the COVID-19 pandemic in Russia, with 98.4% of births having occurred in the hospital setting.

According to a large cohort study of more than 1.6 million pregnant patients across 463 U.S. hospitals, the mode of delivery remained stable during the pandemic with only a marginal relative increase in having a primary CB, a marginal relative decrease in having a repeat CB, and no changes in rates of vaginal, assisted vaginal and vaginal birth after caesarean births (VBACs), in comparison to pre-pandemic period (53). However, in our study we saw significant changes in modes of delivery rates during the pandemic, with significantly fewer emergency CBs and assisted vaginal births and almost a double increase in planned CBs. These findings seem to be contradictory to conclusions of previous studies, which found that greater stress in the moments before delivery could increase the likelihood of needing an instrumental delivery (54). Interestingly, in our study with the growth in the number of planned CBs we also found that women were less satisfied with such birth experiences, which was not the case before the pandemic. A possible explanation for more planned CBs may be the hospitals' staff shortages and preference for scheduled CBs rather than spontaneous vaginal deliveries, which may have been organised against women's original childbirth plans, leading to their disappointment with the childbirth experience. Other possible factors that may explain this increase are the COVID-19 related restrictions against the presence of support persons during labour. We have previously shown that women with accompaniment had fewer CBs in comparison to those giving birth without support in Russia (34, 55). Furthermore, in Russia, regardless of the caesarean surgery being urgent or planned, women always have to stay in an intensive care unit separated from their newborns for a certain period of time, which might have been prolonged during the pandemic, leading to lower satisfaction with the childbirth experience, which is supported by findings of Bryaton and colleagues, who showed that being with their infant the moment after delivery had a greater positive impact on the perceived birth experience than the mode of birth (56). Robbie Davis-Floyd's findings, based on interviews with 165 childbearers, are quite similar; she shows that the joy of holding their newborns skin-to-skin immediately after birth tended to override any negative feelings about the mother's childbirth experiences, at least in the short term (57). However, in the long term after birth, mothers who experienced what Davis-Floyd calls “cognitive dissonance” between their own ideologies around labour and birth and the ideology dominant in U.S hospitals, which Davis-Floyd terms “the technocratic model of birth,” often suffered from postpartum depression or PTSD (57).

The evidence on biomedical interventions during the pandemic is contradictory. Verhoeven and colleagues report a decrease in episiotomy use during the pandemic, but an increase in the use of epidural analgesia use (44). The data from Italy documents the increasing rates of active medical interventions during the COVID-19 pandemic (39).The Canadian study reports a decrease of nitrous oxide and general anaesthesia during the pandemic (45). Our results show consistently high levels of labour medicalisation both before and during the pandemic. However, the number of biomedical interventions and particular types of intervention (episiotomy, synthetic oxytocin, epidural analgesia) were associated with lower birth satisfaction scores only during the pandemic. Thus, although the frequency of biomedical interventions did not increase, women's perceptions of medical assistance have changed. This might be an indication of the growing trend against the biomedicalization of labour (42, 57, 58). The information about risks and benefits of the medical procedures become more available, partly due to the popularity of antenatal education classes (59). There is a growing patients' request for participation in the decision making during labour, including informed consent about any medical interventions (60). Our previous data showed that medical interventions are frequently performed without women's consent, which can be a serious dissatisfaction and traumatic factor (55).

Support during labour has been consistently linked with better birth experiences (21–24, 57) and our study findings from both cohorts corroborate the importance of social support as women who had a partner, a private midwife/doula, or both present during labour and birth reported significantly higher satisfaction with their childbirth experiences than those giving birth in the absence of companions. Yet many hospitals restricted the number of companions allowed during birth as the first line of defence against the spread of the COVID-19 infection. These restrictions were some of the main concerns mentioned by pregnant women during the pandemic who feared that they would give birth alone or without the persons they were expecting to accompany them in Italy (61) and the United States (29). For the majority of women in Russia these fears were fulfilled, as in our study over 70% of women had no companion, and only 4% could have both a partner and doula/private midwife present during labour and birth. While in the Russian birth culture women commonly gave birth without any assistance in the public hospital before the pandemic as well, there was an increasing trend toward having a support person during labour (62). Yet, during the pandemic in the vast majority of hospitals in Russia, the partner either was not allowed to be present, or the conditions for his presence were not realistic, with even fewer hospitals allowing a hired companion. Importantly, while in the majority of countries the restrictions for support person's attendance of labour were lifted as soon as possible (63), in most of Russian federal districts they remained in place regardless of other COVID-19 related measure changes (33). At the same time, Russian women who gave birth with a doula or a private midwife had the highest birth satisfaction scores both before and during the pandemic, which is in line with the study by Hodnett and colleagues where continuous support during labour provided by a woman from outside the hospital or the pregnant woman's social network, e.g., a doula, was associated with health benefits for women and their children (24). Intriguingly, if before the pandemic there was almost no difference in birth satisfaction when giving birth alone or with a partner, during the pandemic Russian women who had a chance to have at least a partner present rated their satisfaction with their birth experiences higher than those with no support. This may indicate that during stressful times women are particularly vulnerable and benefit from any type of continuous support during labour. Therefore, having the right to have a birth team of choice is essential for higher birth satisfaction and better health outcomes for mothers and their infants (24).

Antenatal education is widely popular in Russia, and the majority of women tend to prepare for childbirth, using various strategies (59). In our study, 75% of participants reported following at least one type of childbirth preparation both before and during the pandemic. There is evidence documenting that women intend to get more control and protection during labour and have more positive birth experiences with the help of childbirth preparation (59). This tendency was reinforced during the COVID-19 pandemic, and the frequency of self-education has increased. The perspective of support restriction and infant separation in case of a positive COVID-19 test could also encourage this tendency (33, 64). Our data show that antenatal education is not directly associated with higher birth satisfaction scores. The type of childbirth healthcare plan and support during labour seem to be more significant for a positive birth experience. Sadly, women's expectations and wishes concerning childbirth are often ignored by the healthcare providers (65, 66). Furthermore, it might be difficult to apply the knowledge assimilated during the preparation courses without support in the hospital.

One more possible explanation of these findings is the variety of antenatal education courses. In this study we did not investigate the contents of different birth preparation courses, yet these could vary tremendously from hospital-based classes to traditional “women circles”, providing evidence-based information or ethnoscience (59). The type of childbirth preparation practices and their contents could be the potential area for further research.

## 5. The strengths and limitations of our study

Strengths of our study include the substantial sample sizes of the two cohorts, use of validated psychometric tools, and thorough investigation of birth experiences. Moreover, the inclusion of the questions regarding the COVID-19 diagnosis before, during and after childbirth allows us to evaluate its prevalence and direct effect on women's birth satisfaction. However, several limitations should be addressed when interpreting our results. First, the data for both cohorts was collected anonymously and exclusively online, which may impact the reliability of the responses. Second, all the data are based on self-reports, with no medical records or other objective information on the participants' health and obstetric history, which may limit its validity. Another limitation that should be considered is the unidimensional nature of the BSS-RI measurement of birth satisfaction (67). It presents the possibility of missing important factors affecting women's attitudes toward their childbirth experiences and, thus, the short- and long-term effects of these experiences on their physical and mental health as well as the health of their children. Finally, the majority of the respondents come from big Russian cities, which may limit the generalisability of our results, as practices in maternity care hospitals vary across the country. Hence, further studies using a birth satisfaction instrument that provides ways to evaluate wider aspects of birth experiences or qualitative methods with participants from a wider range of regions of the Russian Federation are warranted.

## 6. Conclusions

Taken together, the results from this study shed light on how the measures that were introduced to protect women, their children, and healthcare workers against the spread of COVID-19 infection had a significant impact on birth experience and birth satisfaction in Russian women. Particularly, lack of respect for the preferred type of childbirth healthcare plan, mode of birth, and amount of medical procedures, as well as lack of support during labour were associated with notably lower birth satisfaction. Thus, these findings provide further support for current 2018 WHO guidelines for intrapartum care (13) on the importance of making childbirth a positive experience for women. Promoting maternal emotional wellbeing alongside physical safety during and after childbirth is of paramount importance. Having the right to have a birth team of choice and respect for the preferred type of childbirth healthcare plan, with the proper legislation and professional support for home birth, are prospective avenues toward increasing birth satisfaction and improving health outcomes for mothers and their infants. This information should be taken into consideration by stakeholders and policy makers in order to estimate risks and benefits when making decisions in ftimes of emergency.

## Data Availability

The raw data supporting the conclusions of this article will be made available by the authors, without undue reservation.
